# Systematic benchmark of single-cell hashtag demultiplexing approaches reveals robust performance of a clustering-based method

**DOI:** 10.1093/bfgp/elae039

**Published:** 2024-10-10

**Authors:** Mohammed Sayed, Yue Julia Wang, Hee-Woong Lim

**Affiliations:** Division of Biomedical Informatics, Cincinnati Children's Hospital Medical Center, 3333 Burnet Ave. Cincinnati OH 45229, United States; Department of Biomedical Sciences, College of Medicine, Florida State University, 1115 W Call St, Tallahassee, FL 32306, United States; Division of Biomedical Informatics, Cincinnati Children's Hospital Medical Center, Department of Pediatrics, University of Cincinnati College of Medicine, 3333 Burnet Ave. Cincinnati OH 45229, United States

**Keywords:** single-cell data, hashtag, demultiplex, benchmark, clustering

## Abstract

Single-cell technology opened up a new avenue to delineate cellular status at a single-cell resolution and has become an essential tool for studying human diseases. Multiplexing allows cost-effective experiments by combining multiple samples and effectively mitigates batch effects. It starts by giving each sample a unique tag and then pooling them together for library preparation and sequencing. After sequencing, sample demultiplexing is performed based on tag detection, where cells belonging to one sample are expected to have a higher amount of the corresponding tag than cells from other samples. However, in reality, demultiplexing is not straightforward due to the noise and contamination from various sources. Successful demultiplexing depends on the efficient removal of such contamination. Here, we perform a systematic benchmark combining different normalization methods and demultiplexing approaches using real-world data and simulated datasets. We show that accounting for sequencing depth variability increases the separability between tagged and untagged cells, and the clustering-based approach outperforms existing tools. The clustering-based workflow is available as an R package from https://github.com/hwlim/hashDemux.

## Introduction

Recent advances in single-cell sequencing technologies [1–7] have opened up the way to study human diseases at a single-cell resolution, allowing researchers to delineate heterogeneous cell status and types. Despite these advances, multisample single-cell experiments are usually conducted in parallel, which involves many challenges such as high cost, batch effects, and unwanted signals from cell multiplets [8]. Sample multiplexing was introduced for higher throughput and effective use of platform capacity, which also alleviates the above challenges. Basically, multiplexing works by giving all cells from a sample a unique barcode, termed a tag, and then pooling all samples for subsequent library preparation and sequencing [9]. Several sample multiplexing technologies have been developed for sample labeling including antibody-based [10], lipid-based [9], and chemical tagging [11]. After sequencing, samples are demultiplexed computationally. Briefly, the tag-by-cell count matrix is generated in addition to the canonical gene-by-cell read counts matrix [12]. The tag counts matrix is subject to computational tools to assign cells to their source sample, i.e. sample demultiplexing. Ideally, the distribution of tag counts across all cells is bimodal with two peaks, representing tagged and untagged cells for a sample, with tagged cells having higher read counts. Subsequently, cells are classified as either tagged with one tag (singlet), tagged with two or more tags (doublets), or not assigned to any tag (negatives). However, real-world cell hashing counts data often suffer from various noises and contaminations, which can lead to the inability to assign cells to their sample of origin. Inaccurate sample demultiplexing is problematic for downstream analysis either by discarding many cells as negatives/doublets or by assigning cells to incorrect samples, which can affect the biological conclusions of downstream analysis [13].

Many demultiplexing tools were developed to tackle this challenge (Table S1). In tag-based demultiplexing approaches, tag count distribution is analyzed individually to classify cells as either tagged (positive) or untagged (negative). Tag-based demultiplexing methods try to find the optimal boundary between the positive and negative populations per tag. For example, deMULTIplex [9] searches for the boundary that maximizes the proportion of predicted singlets. A similar approach is used by Bimodal Flexible Fitting (BFF) [14] but with the boundary set at the minimum density between the two peaks from a bimodal distribution, where it works with either raw counts (bff_raw) or normalized counts (bff_cluster). On the other hand, some tag-based demultiplexing tools fit a statistical model to tag count distribution. For example, GMM_Demux [15] assumes and fits the Gaussian mixture model for positive and negative cells. demuxmix [16] also fits a two-component negative binomial mixture model to raw tag counts, which can utilize the correlation between tag counts and the number of detected genes to increase the performance. Seurat’s HTOdemux [10] fits negative binomial distribution to negative cells only. It identifies negative cells by clustering cells using normalized tag counts and then picking the cluster with the lowest average expression. A recently developed tool, deMULTIplex2 [17], fits two negative binomial generalized linear models to tag counts distributions of negative and positive cells in two different feature spaces and uses total tag counts as a covariate in the model. On the other hand, a cell-based demultiplexing approach classifies cells individually based on the expression of the top two tags, andhashedDrops [18] is an example of such approach.

In practice, tag count data are often noisy, which obscures the separation between tagged and untagged cells. Recent work has shown that although many demultiplexing tools work well for good-quality hashing data, they struggle with low-quality noisy data [12]. There are two main types of technical noises, tag-specific background noise (ambient noise) and cell-specific noise (sequencing depth variability) [17, 19]. Ambient noise is mainly due to the existence of cell-free tags released from stressed or dying cells [20]. Sequencing depth variability happens due to the stochastic nature of library preparation and sequencing. For demultiplexing tools to successfully assign cells to their sample of origin, they must first account for or remove both types of noise. Several methods have been suggested to remove ambient noise from data, including per-tag centered log ratio (CLR) [10], Log2Center [9], and bimodal quantile normalization [14]. On the other hand, sequencing depth per cell often varies by order of magnitude, which makes comparing different tag counts across cells inaccurate. Thus, tag counts are often subjected to normalization such as per-cell CLR [21], relative counts (RC) [22], and log-normalization [22].

Although many existing demultiplexing tools have been recently benchmarked [12], there is no systematic evaluation of the effect of different normalization methods on the sample demultiplexing performance of different tools. Also, one of the intuitive methods, a clustering-based approach, has never been systematically tested despite its versatile potential beyond the cell-type identification and antibody tags data analysis [21]. Here, we perform a comprehensive systematic benchmark of multiple demultiplexing approaches including a clustering-based method in combination with different normalization methods using real-world data and simulated data with varying degrees of noise. In our benchmark, the clustering-based approach displayed the most robust performance.

## Results

### Effects of different types of technical noises on sample demultiplexing

Tag count data for cell hashing suffer from mainly two types of technical noises, i.e. tag-specific background noise (ambient noise) and cell-specific noise (sequencing depth variability) [17, 19]. In this section, we show an example for each type, its impact on sample demultiplexing, and how normalization can mitigate such impact.

**Figure 1:**
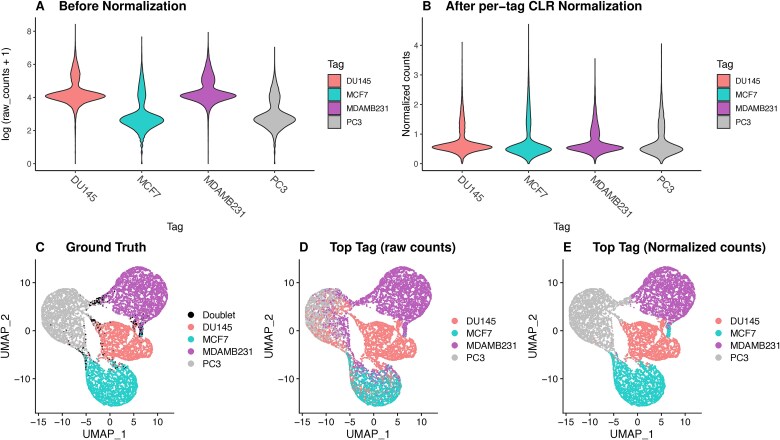
Effect of tag-specific ambient noise on sample demultiplexing in CMO_nuclei dataset [23]. (a) Violin plot of each tag’s counts distribution using raw counts in log scale. (b) Violin plot of each tag’s counts distribution using per-tag CLR-normalized counts. (c) UMAP with cells labeled by ground truth. (d) UMAP with cells labeled by the top expressed tag considering raw tag counts. (e) UMAP with cells labeled by the top expressed tag after normalization and removing tag-specific ambient noise.

Ambient noise denotes the case where some tags display higher baseline expression compared to other tags (Fig. 1a), which means that these tags can be the top expressed tags even in the case of cells stained with other tags (Fig. 1d). This bias makes detected tag counts incomparable, which is particularly problematic for tools that use the highest expressed tags per cell to assign the cell to sample of origin. After applying per-tag CLR normalization (Fig. 1b), tags now have similar baseline expressions, and the top tag per cell (Fig. 1e) is now concordant with ground truth (Fig. 1c).

**Figure 2:**
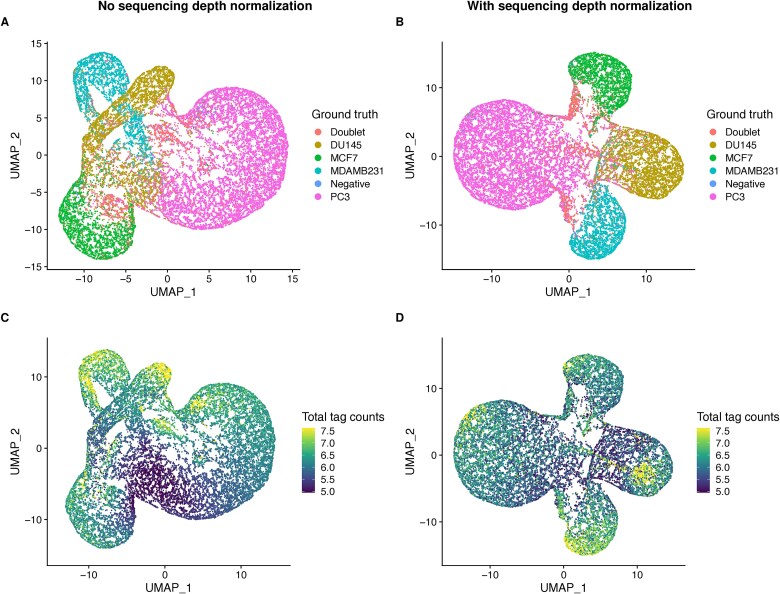
Effect of sequencing depth variability on sample demultiplexing in a dataset with high sequencing depth variability (LMO_custom_cells dataset [23]). (a, b) UMAP of cells colored by ground truth sample classification using either (a) raw tag counts or (b) tag counts after sequencing depth normalization. (c, d) UMAP of cells colored by total tag counts (log scale) and computed using either (c) raw tag counts or (d) tag counts after sequencing depth normalization.

Sequence depth variability is another type of technical noise in cell hashing data due to the stochasticity of sequencing depth per cell. Many state-of-the-art demultiplexing tools assume that sequencing depth variability is insignificant and therefore don’t account for it. Figure 2 shows an example of a dataset with more than two orders of magnitudes in sequence depth variability. The Uniform Manifold Approximation and Projection (UMAP) plot generated without normalizing for sequencing depth shows less separable clusters (Fig. 2a). Also, cells with low total tag counts stained with different tags are clustered together (Fig. 2c). On the other hand, the UMAP generated after normalizing for sequencing depth shows that cells are mainly clustered by sample of origin (Fig. 2b) and less influenced by variability of total tag counts (Fig. 2d).

### Effect of different normalization methods on the separability of positive versus negative cells

Many sample demultiplexing tools aim to find the optimal boundary between two groups of cells, tagged and untagged, in tag count distribution space. The higher separation between the tagged/background cells makes finding the optimal boundary easier and therefore assigning cells to their sample of origin. Given that ground truth is known for the test datasets, we used the area under the precision–recall curve (AUCPR) to quantify the effect of different normalization methods on the separability of these two groups.

**Figure 3:**
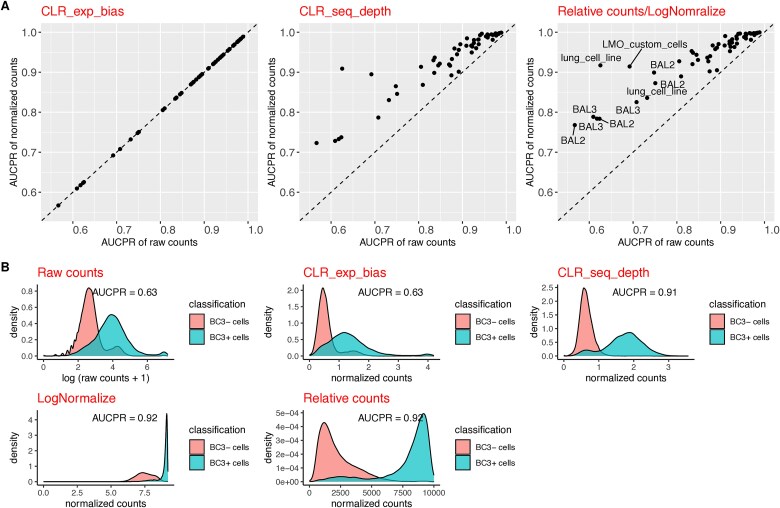
Effects of different tag count normalization methods on separability of tagged/background cells. (a) Tag-level AUCPR values using normalized counts compared to raw counts (for different normalization methods). Each point represents one tag. (b) An example of increased separability after normalizing for sequencing depth. Red and green represent negative and positive cells, respectively, for “BC3” tag from the lung cell line dataset [12].

We measured the separability of tags in 11 real datasets when using four different normalization methods, namely, per-tag CLR (CLR_exp_bias), per-cell CLR (CLR_seq_depth), RC, and log normalization (LogNormalize) (see Methods for a detailed formula). Normalization methods that take into account sequencing depth variability (i.e. CLR_seq_depth normalization, RC, and log normalization) increase the separation between the positive versus negative cells compared to raw counts (Fig. 3a). As expected, CLR_exp_bias normalization didn’t change the separability because it is a monotonic transformation, i.e. does not change the order of cells. Tags from BAL2, BAL3, lung_cell_line, and LMO_custom_cells datasets have benefited the most from sequencing depth normalization. Two datasets (BAL2 and BAL3) are low-quality cell hashing data [12]. An example of one of these tags (BC3 from lung_cell_line dataset) where separability increased by at least 45% with sequencing depth normalization (Fig. 3b).

### Benchmarking of normalization-based demultiplexing workflows on real datasets

**Figure 4:**
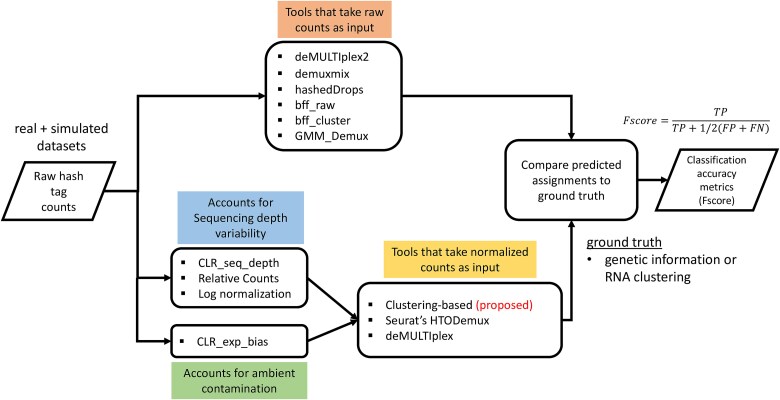
Overall workflow for the benchmarking of different combinations of normalization methods and demultiplexing tools.

Next, we assessed how different demultiplexing methods can benefit from the increased separability and compared their demultiplexing performance to current state-of-the-art demultiplexing tools. We compared the demultiplexing performance of 18 different demultiplexing workflows (12 normalization-based + 6 standalone tools as shown in Fig. 4 and Table S1). Normalization-based workflows include all combinations of four different normalization methods and three tools that take normalized tag counts as an input (Seurat’s HTODemux [10], MULTISeq, which is Seurat’s implementation (“MULTIseqDemux” function) of deMULTIplex [9], and clustering-based demultiplexing). Standalone tools include deMULTIplex2 [17], demuxmix [16], GMM_Demux [15], hashedDrops [18], and two modes of BFF method (bff_raw and bff_cluster) [14]. All tools were run using default parameters.

**Figure 5:**
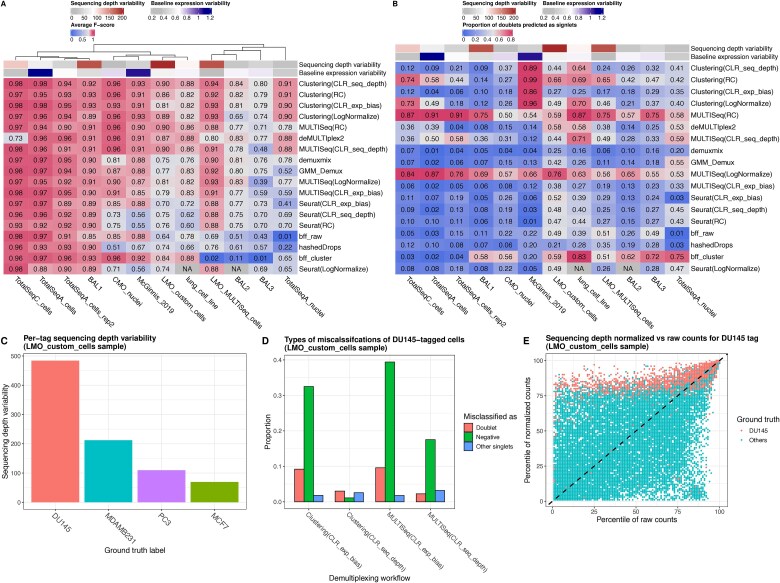
Performance on real datasets. Heatmaps of (a) average F-score and (b) proportion of doublets misclassified as singlets for each demultiplexing workflow (rows) on 12 real datasets (columns). Normalization-based workflows are denoted as “tool(normalization method).” Rows are ordered by average F-score across all datasets. NA indicates a failed run. For each dataset, we added a column annotation indicating the level of baseline expression variability and sequencing depth variability. (c) Per-tag sequencing depth variability. (d) Proportion of different types of misclassifications of DU145-tagged cells for clustering-based and MULTIseq-based demultiplexing workflows with and without sequencing depth normalization. (e) Percentile of each cell’s DU145 tag’s CLR_seq_depth-normalized counts versus raw counts. (c–e) are from LMO_custom_cells sample.

We performed a comprehensive benchmark of the 18 demultiplexing workflows using 12 real-world datasets (Table S2) with known ground truth. Of note, these test data sets are based on more than one type of cell hashing biochemistry. Remarkably, clustering-based workflows displayed robust performance in most datasets and were less sensitive to the choice of the normalization method (Fig. 5a). Among clustering-based workflows, per-cell CLR normalization (CLR_seq_depth) displayed superior performance in almost all datasets (Fig. 5a). On the other hand, Seurat and MULTISeq tools were more sensitive to the choice of the normalization method. MULTISeq performed well when combined with sequencing depth normalization. Seurat showed better performance when correcting for the variability of per-tag ambient noise using per-tag (CLR_exp_bias) normalization, especially for datasets with high values of baseline expression variability, e.g. McGinnis_2019 and CMO_nuclei where the F-score of Seurat workflow utilizing per-tag CLR normalization has increased by at least 57% and 16%, respectively, compared to Seurat workflow utilizing sequencing depth normalization (Fig. 5a).

To understand how accounting for sequencing depth variability improves demultiplexing accuracy, especially in datasets with high variability of sequencing depth, we focused on one dataset (LMO_custom_cells) [23] with the largest variability in total tag counts. Workflows that account for sequencing depth such as Clustering (CLR_seq_depth), MULTISeq (CLR_seq_depth), and MULTISeq (RC) were the best performers (average F-score = 0.9). Also, deMULTIplex2 performed reasonably well (F-score = 0.87) as shown in Fig. 5a. DU145-tagged cells have relatively higher variability relative to other samples (Fig. 5c). It has been reported previously that a large proportion of DU145-tagged cells tend to be misclassified as negatives [23]. Utilizing sequencing depth-normalized (per-cell CLR-normalized) counts, compared to per-tag CLR normalized counts, recovered ~97% and ~56% of cells misclassified as negatives in the case of clustering-based and MULTISeq-based workflows, respectively (Fig. 5d). As shown in Fig. 5e, sequencing depth-normalization boosts up the rank of DU145-tagged cells with low raw counts, thereby making them less likely to be misclassified as negatives.

Another metric to measure the demultiplexing performance is doublet calling [12]. Figure 5b shows a heatmap with the proportion of “true” doublets, based on ground truth, misclassified as singlets. Doublet-to-singlet misclassifications are particularly important because they can be problematic for downstream analysis [24]. Most workflows have a low misclassification rate in most datasets. Although sequencing depth normalization followed by MULTISeq showed better performance in calling singlets, their doublet calling was the worst, with MULTISeq(RC) and MULTISeq(LogNormalize) misclassifying at least half of the doublets as singlets in all datasets. Notably, the clustering-based methods displayed high misclassification rates for one data set, McGinnis_2019. This can be attributed to the small number of ground truth doublets (98 cells) and the fact that clustering-based demultiplexing can miss such cells if they don’t form a separate cluster but are part of bigger clusters. Also, it should be noted that the ground truth doublets in these particular data sets were defined by RNA-based doublet prediction. Meanwhile, the impact of the missed doublets in the downstream analysis should be minimal since it would happen when the number of doublets is extremely small relative to the whole population; those doublets could be cleaned up by using existing RNA-based doublet detection tools such as scDblFinder [25] and Scrublet [24] after sample demultiplexing-based doublet calling. Indeed, when we ran the scDblFinder doublet detection tool on this dataset, we were able to detect 97% of doublets.

### Robustness of demultiplexing workflows against high background noise

It has been shown here and previously [12] that most demultiplexing workflows work well in the case of good-quality cell hashing data; therefore, we wanted to assess their performance in the case of low-quality data with high background noise. We generated 120 5-tag simulated datasets of varying levels of the two types of contamination, ambient and cell-bound, to assess the robustness of the demultiplexing performance. Contamination level parameters were chosen to simulate the high-background noise scenarios beyond the 12 real datasets used in this study as shown in Supplementary Fig. S1.

**Figure 6:**
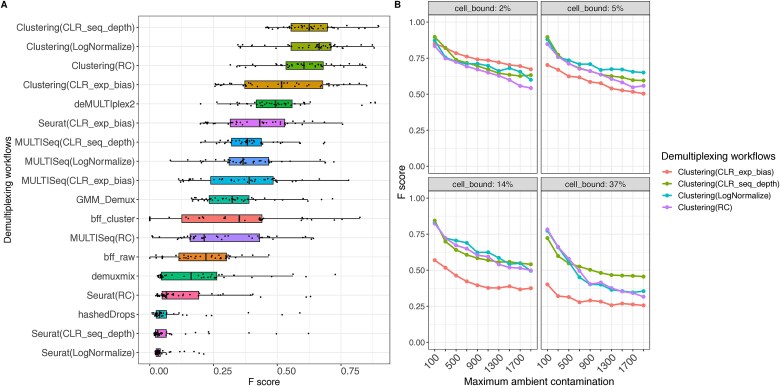
Performance on high-background noise simulated datasets. (a) Distribution of F-scores of each workflow across datasets. Workflows are ordered by average F-score across all datasets. (b) Dissection of clustering-based demultiplexing performance by different types of contamination.

Overall, clustering-based workflows displayed the highest average F-scores when combined with normalization methods that address sequencing depth variability (Fig. 6a). On the other hand, BFF, demuxmix, hashedDrops, Seurat-based workflows with sequencing depth normalization, and MULTISeq with RC normalization were less robust in case of high background noise (Fig. 6a). Then, we assessed the effect of each type of contamination on demultiplexing performance. The results in Fig. 6a were separated by the levels of ambient and cell-bound contaminations. In general, the performance of all workflows degrades with the increase of contamination level. As expected, per-tag CLR (CLR_exp_bias) normalization was the best performer in case of the presence of high levels of ambient contamination and very low cell-bound contamination (~2% of total cell-bound reads) (Fig. 6b). On the other hand, normalization methods that take into account sequencing depth variability have better performance in case of higher levels of cell-bound contamination with per-cell CLR (CLR_seq_depth) showing higher robustness to higher levels of both types of contamination (Fig. 6b).

Lastly, to assess how the run time of clustering-based demultiplexing workflow is compared to other workflows with increasing number of tags (samples) and number of cells per tag, we generated simulated data that have a number of tags equal to one of the following values {5, 10, 20} and a number of cells per tag is one of the following values {100, 200, 400, 800}. In general, clustering-based workflow shows comparable run time with other state-of-the-art tools as shown in Supplementary Fig. S2. Some workflows, e.g. Seurat, MULTISeq, and demuxmix, were very fast regardless of the size of the dataset or the number of tags. On the other hand, some workflows, e.g. clustering-based, deMULTIplex2, bff_cluster, bff_raw, and hashedDrops, showed a significant increase in run time with an increasing number of tags and/or number of cells per tag.

## Discussion

Sample multiplexing has provided a cheap and scalable solution for researchers to conduct multisample single-cell experiments. It relies on computational tools to assign cells to their sample of origin. Real-world cell hashing data suffer from different types of technical noise, mainly ambient contamination and sequencing depth variability. Despite their importance, there has been no systematic benchmark of the tag count normalization methods and their effect on demultiplexing performance. First, we compared four normalization methods: three accounting for sequencing depth variability (RC, LogNormalize, and per-cell CLR) and one accounting for ambient contamination (per-tag CLR). We found that normalization methods that account for sequencing depth variability increase the separation between positive and background cells, especially for low-quality data. RC and log normalization provide slightly better separation than per-cell CLR. The increased separation can enhance the accuracy of the subsequent demultiplexing step, especially for tools that search for the optimal boundary between tagged and untagged cells in tag counts histogram, e.g. MULTISeq. In line with these results, a workflow that uses RC-normalized counts followed by MULTISeq showed better performance compared to the standard MULTISeq workflow that only accounts for ambient contamination.

Next, we benchmarked 12 normalization-based demultiplexing workflows and compared their performance to six additional standalone tools using real and simulated datasets with ground truth labels. In addition to existing tools, we tested a clustering-based demultiplexing workflow similar to the one used for CITE-seq data [21]. This workflow starts with clustering of cells using normalized tag counts and then differential tag expression analysis is performed to assign cells to their original sample. Interestingly, we found that clustering-based workflows, regardless of the normalization method, have the best demultiplexing performance. Among these, the workflow that uses per-cell CLR normalization was the top performer. The robust performance of this workflow is thought to be attributed to the utilization of the similarity of the normalized tag counts rather than the explicit modeling of assumed noise. In addition, in each cell, tag counts can be treated as part of a whole (i.e. compositional data) [21,26,27], and therefore, CLR normalization performs better than RC or log-normalization for sequencing depth normalization. Although some real datasets can have a significant difference in total tag counts across cells, most widely used tools account only for ambient contamination levels. A recently developed tool, deMULTIplex2, accounts for both types of contamination, but it assumes that sequencing depth variability is expected to be less than two orders of magnitude [17]. This may justify the decrease in performance in datasets that violate this assumption, e.g. LMO_custom_cells, LMO_MULTISeq_cells, and TotalSeqC_cells datasets. A comparison of key strengths, weaknesses, and recommended normalization methods is summarized in Table 1.

**Table 1:** Comparison of the performance of different sample demultiplexing workflows and their strengths and weaknesses

**Tool**	**Current normalization**	**Recommended normalization**	**Strengths**	**Weaknesses**
Clustering-based	–	Per-cell CLR normalization	Accounts for both types of contamination Robust to high background noise	Sensitive to clustering parameters
deMULTIplex (MULTIseq)	Per-tag CLR normalization	Relative counts/per-cell CLR normalization	Very fast Accounts for both types of contamination	Fails when tag count distribution isn’t bimodal
Seurat	Per-tag CLR normalization	Per-tag CLR normalization	Very fast Accounts for ambient noise	Doesn’t account for sequencing depth variability
GMM_Demux	Per-tag CLR normalization	–	Accounts for ambient noise	Doesn’t account for sequencing depth variability Fails when tag count distribution isn’t bimodal
demuxmix	Raw counts	–	Very fast Accounts for ambient noise	Doesn’t account for sequencing depth variability Fails when tag count distribution isn’t bimodal
deMULTIplex2	Raw counts	–	Accounts for both types of contamination	Suboptimal performance when sequencing depth variability is larger than two orders of magnitude
bff_raw	Raw counts	–	Accounts for ambient noise	Doesn’t account for sequencing depth variability Fails when tag count distribution isn’t bimodal
bff_cluster	Bimodal quantile normalization	–	Accounts for ambient noise Optimized for tag count distributions with clear Bimodality	Doesn’t account for sequencing depth variability Fails when tag count distribution isn’t bimodal
hashedDrops	Ambient-corrected raw counts	–	Accounts for ambient noise No assumptions about tag count distribution	Parameters need to be tuned for each dataset

To test the robustness of different workflows in the case of highly noisy datasets, we generated simulated datasets with varying levels of the two types of contamination, ambient and cell-bound. Similar to their performance in real datasets, clustering-based workflows displayed robust performance. On the other hand, some tools including BFF, demuxmix, and MULTISeq-based workflows performed poorly, mainly because data high-background noise violates their bi-modality assumption of tag count distribution.

Despite the robust performance of the clustering-based approach, they have some limitations, including its sensitivity to the choice of the parameters controlling the granularity level of clustering. Especially, given the nature of the clustering algorithm relying on nearest neighbors, samples with a too-small number of cells [below *k* in *k*-nearest neighbor (KNN)] are more susceptible to error. In this case, it is better to increase the granularity level of clustering, i.e. decreasing the *k* parameter of the KNN algorithm to be less than the expected minimum number of cells per sample and/or increase the resolution parameter. In addition, the doublet calling of the clustering-based workflow was suboptimal. Thus, the clustering-based methods would benefit from *post hoc* doublet filtering based on RNA data.

In this study, we used 12 real datasets to benchmark different demultiplexing workflows. Although these datasets represent different cell hashing biochemistries and levels of contamination (Table S2), they have a limited number of tags (the maximum number of tags per dataset was 12), and many of the multiplexed samples are cell lines. In the future, datasets with larger-scale multiplexed samples and with heterogeneous cell types will allow us to assess the performance of different demultiplexing workflows in more complex scenarios.

## Conclusions

In summary, we showed that accounting sequencing depth variability increases the separability between tagged and background cells and subsequently can enhance the performance of existing demultiplexing tools like MULTISeq (deMULTIplex). Benchmarking indicates that per-cell CLR normalization followed by clustering-based demultiplexing has better performance compared to state-of-the-art tools, even in the presence of high background noise. Clustering-based workflow is available as an open-source R package.

## Methods

### Normalization methods of tag counts data

Assume that $R$ is $M$ tags $\times N$ cells matrix representing raw tag counts matrix and *r_i,j_* is the number of reads of tag*_i_* in cell*_j_*; then, normalized counts ${n}_{i,j}$ are computed as follows for each normalization method:

Log normalization (LogNormalize)


\begin{align*} {n}_{i,j}=\log \left(\frac{r_{i,j}\times{10}^4}{S_j}+1\right) \end{align*}

where *S*_*j*_ is the sum of the counts of all tags in *cell*_*j*_ and $\log$ is the natural logarithm.

Relative Counts (RC)


\begin{align*} {n}_{i,j}=\frac{\left(\ {r}_{i,j}\times{10}^4\right)}{S_j} \end{align*}

where *S*_*j*_ is the sum of the counts of all tags in *cell*_*j*_.

Per-tag centered log ratio (*CLR_exp_bias*)


\begin{align*} {n}_{i,j}=\log \left(\frac{r_{i,j}}{G_i}+1\right) \end{align*}

where ${G}_i$ is the geometric mean of ${tag}_i$counts in $\mathrm{all}\ \mathrm{cells}$.

Per-cell centered log ratio (*CLR_seq_depth*)


\begin{align*} {n}_{i,j}=\log \left(\frac{r_{i,j}}{G_j}+1\right) \end{align*}

where ${G}_j$ is the geometric mean of the counts of all tags in *cell*_*j*_.

We used Seurat’s *NormalizeData* function to run all normalization methods.

### Clustering-based sample demultiplexing

To cluster cells based on normalized tag counts, we used a workflow similar to the one for proposed for antibody counts [21]. We mainly used the Seurat single-cell data analysis toolkit [28] to implement this workflow as shown in Supplementary Fig. S3. Briefly, we used *FindNeighbors* function to construct a shared nearest neighbor graph and then used *FindClusters* function to run the Louvain method for community detection [29]. To find marker tags per cluster, we ran the Wilcoxon rank sum test using *FindAllMarkers* function. A log fold-change (LogFC) threshold that yields a doublet rate closest to the expected doublet rate is picked. The expected doublet rate can be set by the user or automatically estimated using the number of cells, number of tags, and cell multiplets rate for standard 10× Genomics assay (3′ v3.1) using the following equation [30]:


\begin{align*}& Expected\ doublet\ rate=\\&8\times{10}^{-6}\times \frac{Number\ of\ cells\times \left( Number\ of\ tags-1\right)}{Number\ of\ tags} \end{align*}

If multiple values fit the above criteria, the smallest logFC value is chosen. Only markers that meet the following criteria are kept: (i) a marker tag for a cluster should be expressed in all cells in the cluster and (ii) adjusted *P*-value <.05. Cells are labeled as follows: cells that belong to clusters with no marker tags are labeled as “Negatives,” the ones that belong to clusters with more than one marker tags are labeled as “Doublets,” and the rest are labeled to the corresponding marker tag.

The results of the clustering-based demultiplexing workflow are sensitive to two parameters: the *k* parameter of the KNN algorithm and the resolution parameter in the Louvain community detection method. To avoid this issue, the clustering step is run multiple times, each with different combinations of *k* and resolution values, and then, we use a majority vote to pick a label. *k* and resolution ranges of values (*k* ∈{5,10,15,20,25,30} and resolution ∈{1,2,3,4}) were picked to ensure over-clustering to capture all possible clusters of cells, i.e. cells labeled with one tag, in addition to cells labeled with different combinations of tags. Also, running a clustering-based demultiplexing workflow multiple times allows us to compute a confidence/robustness score, i.e. the proportion of times we see the majority-voted label. This confidence score can be used for additional filtering.

### Real datasets

We compiled 12 publicly available datasets (Table S2) with different levels of quality, types of contamination, number of tags, number of cells, biological samples, and hashing technology (antibody-, lipid-, or cholesterol-based) along with their ground truth [9, 12, 23]. Ground truth provides a “true” label for each cell based on genetic variation or RNA clustering. For each dataset, we computed two values, baseline expression variability (ambient noise) and sequence depth variability, to quantify the two types of background noise as follows:


\begin{align*} &Baseline\ expression\ variaility=\\&\frac{standard\_ deviation\left( med\_ expression\_ tags\right)\ }{mean\left( med\_ expression\_ tags\right)} \end{align*}

where


\begin{align*} &med\_ expression\_ tags=\\&\left\{ med\_ expression\_{tag}_1, med\_ expression\_{tag}_2,\dots \right\} \end{align*}

$med\_ expression\_{tag}_i= median\left(\ \log \Big({tag}_i\_ counts+1\right) \Big)$ , i $\in \left\{1,2,\dots, number\_ tags\right\}$


\begin{align*} & Sequence\ depth\ variability=\\& \frac{99^{th}\ \mathrm{percentile}\ \mathrm{of}\ \mathrm{total}\ \mathrm{tag}\ \mathrm{counts}\ \mathrm{across}\ \mathrm{all}\ \mathrm{cells}}{1^{st}\ \mathrm{percentile}\ \mathrm{of}\ \mathrm{total}\ \mathrm{tag}\ \mathrm{counts}\ \mathrm{across}\ \mathrm{all}\ \mathrm{cells}} \end{align*}

### Simulated datasets with high background noise

We used a modified version of deMULTIplex2’s simulateTags function [17] to generate the simulated data. The function was modified to guarantee that one of the barcodes’ ambient contamination level is equal to the “*max.ambient.log*” parameter and that other barcodes’ ambient contamination levels are uniformly sampled from values between “*min.ambient.log*” and “*max.ambient.log*” parameters. All datasets have five tags, with an equal number of cells per tag (*n* = 1000) and 10% doublet rate. Initially, cells tagged with a specific tag are assigned a random read count between 10 and 1000 for this tag and zero counts for other tags. Then, contamination reads are added as follows: percentage of cell-bound contamination reads equals to one of the following values {2%, 5%, 14%, 37%}. On the other hand, maximum ambient read counts range from 100 to 1900, with step size = 200 (10 different values). Using these parameters, we generated 120 total datasets (40 datasets with different combinations of contamination levels multiplied by three replicates).

### Computational performance analysis

We used the modified simulateTags function in deMULTIplex2 [17] to generate the simulated datasets with varying numbers of tags and number of cells per tag. Generated data have a number of tags equal to one of the following values {5, 10, 20, 40} and the number of cells per tag is one of the following values {100, 200, 400, 800}. Initially, cells tagged with a specific tag are assigned a random read count between 10 and 1000 for this tag and zero counts for other tags. Simulated datasets were generated with low contamination reads (~2% cell-bound contamination reads and the maximum number of ambient reads = 100) and 10% doublet rate. We compared the run time for different demultiplexing workflows on a high-performance computing system. For normalization-based workflows (i.e. clustering-based, Seurat, and MULTISeq), only the best-performing combination with a normalization method is included.

### Separability analysis

We used the AUCPR to evaluate the separability of positive and negative classes. The AUCPR is a suitable metric for the evaluation of binary classifiers when the proportions of positive and negative instances are not equal (imbalanced dataset), which is the case with sample demultiplexing datasets where there are typically more than two samples, and the proportion of tagged cells are much less than the untagged ones. To estimate the AUCPR, the precision–recall curve needs to be constructed with precision on the *y*-axis and recall on the *x*-axis. Each point in the curve corresponds to a threshold in the tag count space that separates positive (tagged) from negative (untagged) cells. For each threshold, precision and recall are calculated as follows:


\begin{align*} Precision=\frac{True\ positives\ }{True\ positives+ False\ positives\ } \end{align*}

\begin{align*} Recall=\frac{True\ positives\ }{True\ positives+ False\ negatives\ } \end{align*}

where ‘true positives’ are the number of positive (tagged) cells with tag count larger than the threshold, ‘false positives’ are the number of negative (untagged) cells with tag count larger than the threshold, and ‘false negatives’ are the number of positive (tagged) cells with tag count smaller than the threshold.

The separability of the two tag count distributions for positive and negative cells for each tag was quantified by computing the AUCPR value using *pr.curve* function from *PRROC* R package [31]. To define positive and negative cells, first, ground-truth doublet cells were excluded, and then, positive cells for a specific tag were defined as cells labeled with this tag, and all other cells were labeled as negative cells. For each tag, higher AUCPR values indicate higher separability.

## Supplementary Material

Supplementary_elae039

## Data Availability

Raw tag counts matrices for real datasets and their ground truth are publicly available. Cancer cell line sample multiplexing datasets (TotalSeqA_cells, TotalSeqA_cells_rep2, TotalSeqC_cells, LMO_MULTISeq_cells, LMO_custom_cells, CMO_nuclei and TotalSeqA_nuclei) from Mylka *et al.*, (2022) [23] can be downloaded from European Nucleotide Archive under accession number E-MTAB-9964. The three batches of BAL (BAL1, BAL2, and BAL3) and lung cell line datasets from Howitt *et al.* [12] can be downloaded from https://github.com/Oshlack/hashtag-demux-paper. The mixed human/mouse dataset (McGinnis_2019) from McGinnis *et al.* [9] was downloaded from https://www.nature.com/articles/s41592-019-0433-8#Sec31 (source data for Fig. 1).

## Code availability

The code to replicate the benchmark in this paper can be found at https://github.com/hwlim/scDemuxBench. Clustering-based sample demultiplexing tool is available as an R package from https://github.com/hwlim/hashDemux.

## Notes

**Mohammed Sayed** is a postdoctoral research fellow in the Division of Biomedical Informatics at Cincinnati Children’s Hospital Medical Center. He received his PhD degree in Computer Science and Engineering from the University of Louisville in 2021. His research focuses on developing computational tools for single-cell sequencing data analysis.

**Hee-Woong Lim** is an assistant professor in the Division of Biomedical Informatics, Cincinnati Children's Hospital Medical Center, Department of Pediatrics, University of Cincinnati College of Medicine. After receiving a PhD in Computer Science and Engineering at the Seoul National University, Republic of Korea, he got bioinformatics postdoctoral training at the University of Pennsylvania. As a bioinformatics scientist, he is interested in understanding the fundamental gene transcriptional regulations in various biological contexts.

**Yue Julia Wang** is an assistant professor in the Department of Biomedical Sciences at Florida State University College of Medicine. She received her PhD in Human Genetics at Johns Hopkins University and did her postdoctoral training at the University of Pennsylvania. She applies highly innovative single-cell multiomics as well as functional genomics approaches to elucidate pathways involved in endocrine cellular plasticity and diabetes pathogenesis.
