# Supplementary material for: Systematic benchmark of single-cell hashtag demultiplexing approaches reveals robust performance of a clustering-based method
Source: Brief Funct Genomics. 2024 Oct 10;24:elae039. doi: 10.1093/bfgp/elae039 (PMC11735735; doi:10.1093/bfgp/elae039)
Supplement: Supplementary_elae039 [file supplementary_elae039.pdf]

Table S1. Sample demultiplexing tools used for benchmarking

Tool	Input	Detailed approach	Assumptions	Reference
deMULTIplex	log2Center normalized counts	For each normalized tag counts distribution, find classification threshold that maximizes singlets between the two peaks	Bimodality of tag counts distribution	McGinnis et al., (2019) [1]
HTOdemux	Per tag normalized counts	Fitting a negative bimodal model to counts of negative cells	-	Stoeckius et al., (2018) [2]
GMM_Demux	Per-tag CLR Normalized counts	Fitting a gaussian mixture model	Bimodality of tag counts distribution	Xin et al., (2020) [3]
demuxmix	Raw counts	Fitting a negative bimodal mixture model	Bimodality of tag counts distribution	Klein (2023) [4]
deMULTIplex2	Raw counts	Fitting two negative bimodal generalized linear models to positive and negative cells in two different spaces	Range of total tag counts is less than two orders of magnitude	Zhu et al., (2024) [5]
bff_raw	Raw counts	Uses a classification threshold that is the local minimum between the two peaks	Bimodality of tag counts distribution	Boggy et al., (2022) [6]
bff_cluster	Bimodal quantile normalization	Uses a threshold that is the local minimum between positive/negative cells peaks	Bimodality of tag counts distribution	Boggy et al., (2022) [6]
hashedDrops	Ambient-corrected raw counts	Droplet classification is determined by checking if log-fold change between the largest and second-largest abundances and second-largest to estimated amount of ambient contamination in that drop exceed some thresholds	-	Lun et al., (2019) [7]

Table S2. Real benchmarking datasets

Dataset	Cell type	Tagging technology	Ground truth	# of tags	Reference
BAL1	Broncho-alveolar lavage fluid	Antibody	Genotyping	8	Howitt et. al. (2022) [8]
BAL2	Broncho-alveolar lavage fluid	Antibody	Genotyping	8	Howitt et. al. (2022) [8]
BAL3	Broncho-alveolar lavage fluid	Antibody	Genotyping	8	Howitt et. al. (2022) [8]
lung_cell_line	Lung cancer cell lines	Lipid	Genotyping	3	Howitt et. al. (2022) [8]
TotalSeqA_cells	Cancer cell lines	Antibody	Genotyping	4	Mylka et al. (2022) [9]
TotalSeqA_cells_rep2	Cancer cell lines	Antibody	Genotyping	4	Mylka et al. (2022) [9]
TotalSeqC_cells	Cancer cell lines	Antibody	Genotyping	4	Mylka et al. (2022) [9]
LMO_MULTIseq_cells	Cancer cell lines	Lipid	Genotyping	4	Mylka et al. (2022) [9]
LMO_custom_cells	Cancer cell lines	Lipid	Genotyping	4	Mylka et al. (2022) [9]
CMO_nuclei	Cancer cell lines (nuclei)	Cholesterol	Genotyping	4	Mylka et al. (2022) [9]
TotalSeqA_nuclei	Cancer cell lines (nuclei)	Antibody	Genotyping	4	Mylka et al. (2022) [9]
McGinnis_2019	Mixed human/mouse cells	Lipid and cholesterol	RNA clustering	12	McGinnis et al., (2019) [1]

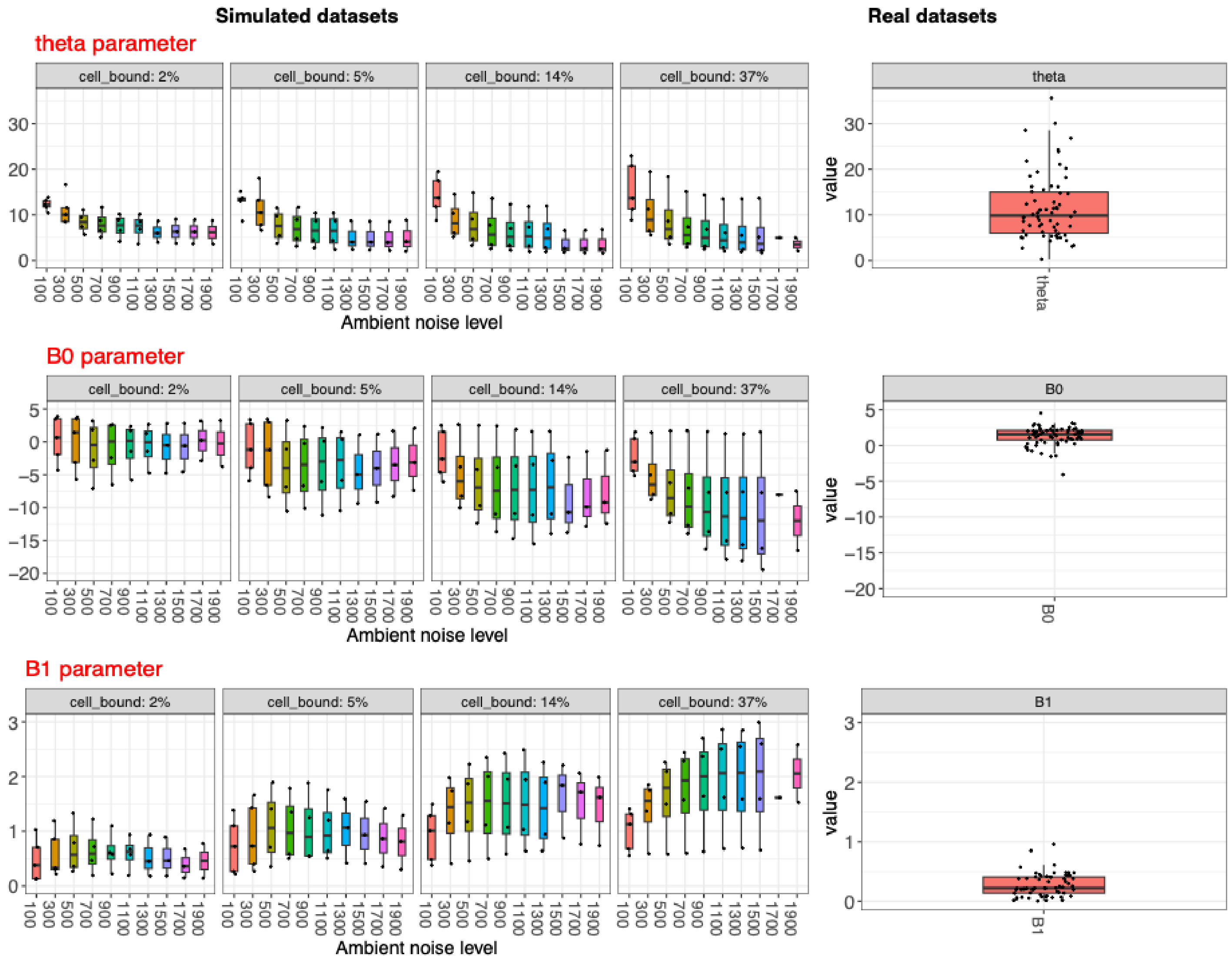

Supplementary Figure S1: Ranges of deMULTIplex2 estimated GLM-NB model parameters for simulated with high-background noise vs real datasets.

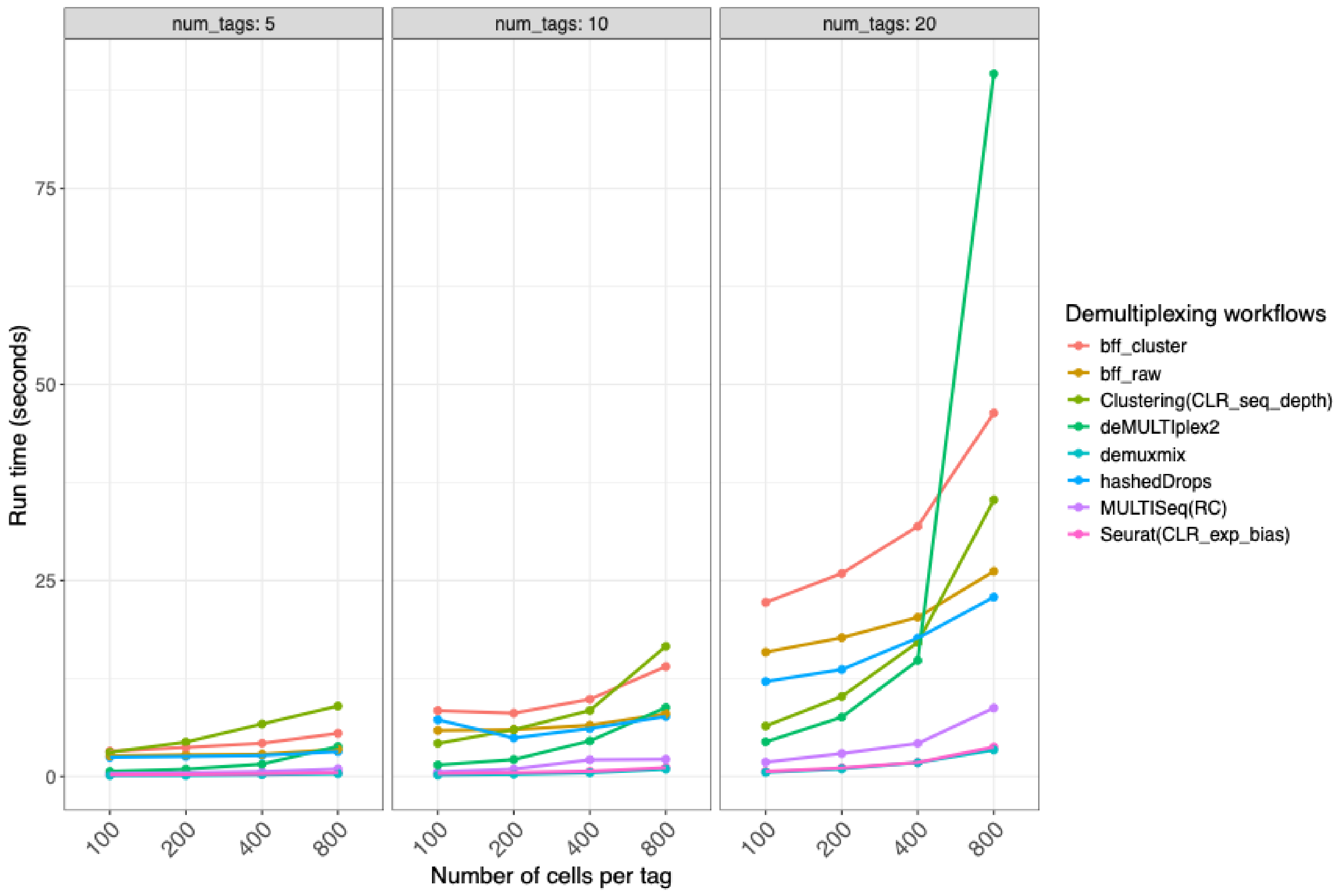

Supplementary Figure S2: Computational performance analysis of different demultiplexing workflows on simulated data. GMM_Demux tool was excluded because it was stuck for several hours.

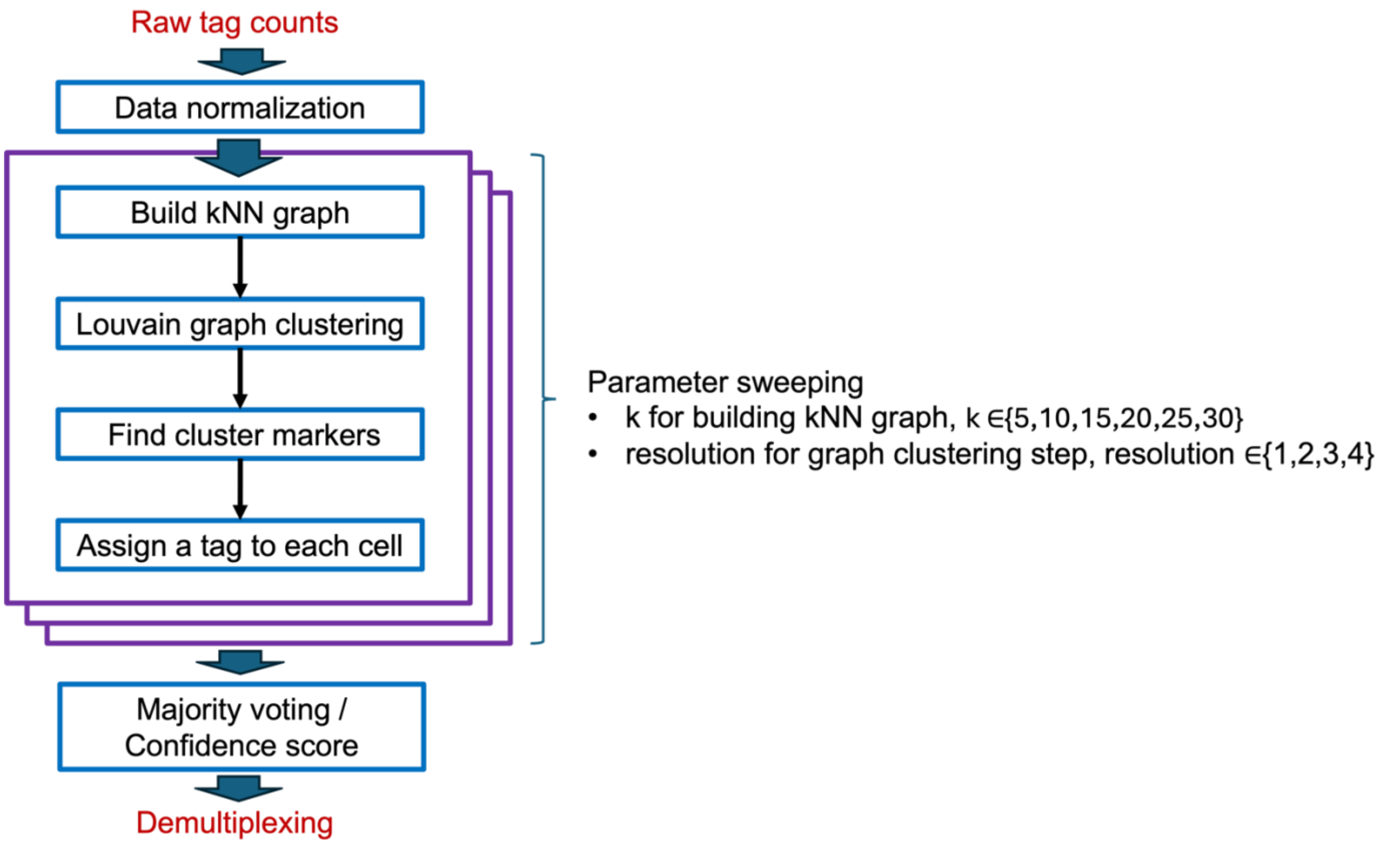

Supplementary Figure S3: Overall workflow of clustering-based sample demultiplexing.

References

1. McGinnis CS, Patterson DM, Winkler J, Conrad DN, Hein MY, Srivastava V, et al. MULTI-seq: sample multiplexing for single-cell RNA sequencing using lipid-tagged indices. Nature methods. 2019;16(7):619-26.
2. Stoeckius M, Zheng S, Houck-Loomis B, Hao S, Yeung BZ, Mauck WM, et al. Cell Hashing with barcoded antibodies enables multiplexing and doublet detection for single cell genomics. Genome biology. 2018;19(1):1-12.
3. Xin H, Lian Q, Jiang Y, Luo J, Wang X, Erb C, et al. GMM-Demux: sample demultiplexing, multiplet detection, experiment planning, and novel cell-type verification in single cell sequencing. Genome biology. 2020;21(1):1-35.
4. Klein H-U. demuxmix: demultiplexing oligonucleotide-barcoded single-cell RNA sequencing data with regression mixture models. Bioinformatics. 2023;39(8).
5. Zhu Q, Conrad DN, Gartner ZJ. deMULTIplex2: robust sample demultiplexing for scRNA-seq. Genome Biology. 2024;25(1):37.
6. Boggy GJ, McElfresh G, Mahyari E, Ventura AB, Hansen SG, Picker LJ, et al. BFF and cellhashR: analysis tools for accurate demultiplexing of cell hashing data. Bioinformatics. 2022;38(10):2791-801.
7. Lun AT, Riesenfeld S, Andrews T, Dao TP, Gomes T, Jamboree PitsHCA, et al. EmptyDrops: distinguishing cells from empty droplets in droplet-based single-cell RNA sequencing data. Genome biology. 2019;20:1-9.
8. Howitt G, Feng Y, Tobar L, Vassiliadis D, Hickey P, Dawson MA, et al. Benchmarking single-cell hashtag oligo demultiplexing methods. NAR Genomics and Bioinformatics. 2023;5(4):lqad086.
9. Mylka V, Matetovici I, Poovathingal S, Aerts J, Vandamme N, Seurinck R, et al. Comparative analysis of antibody-and lipid-based multiplexing methods for single-cell RNA-seq. Genome Biology. 2022;23(1):1-21.
